# A new glimpse of FadR-DNA crosstalk revealed by deep dissection of the *E. coli* FadR regulatory protein

**DOI:** 10.1007/s13238-014-0107-3

**Published:** 2014-10-15

**Authors:** Yongchang Zhang, Rongsui Gao, Huiyan Ye, Qingjing Wang, Youjun Feng

**Affiliations:** 1 College of Life Sciences, Nanjing Normal University, Nanjing, 210023 China; 2 State Key Laboratory for Diagnosis and Treatment of Infectious Diseases, Collaborative Innovation Center for Diagnosis and Treatment of Infectious Diseases, First Affiliated Hospital, Zhejiang University School of Medicine, Hangzhou, 310003 China; 3 Department of Medical Microbiology and Parasitology, Center for Infection and Immunity, Zhejiang University School of Medicine, Hangzhou, 310058 China; 4 College of Life Science and Technology, Guangxi University, Nanning, 530004 China

**Keywords:** FadR, fatty acid metabolism, crosstalk

## Abstract

*Escherichia coli* (*E. coli*) FadR regulator plays dual roles in fatty acid metabolism, which not only represses the fatty acid degradation (*fad*) system, but also activates the unsaturated fatty acid synthesis pathway. Earlier structural and biochemical studies of FadR protein have provided insights into interplay between FadR protein with its DNA target and/or ligand, while the missing knowledge gap (*esp.* residues with indirect roles in DNA binding) remains unclear. Here we report this case through deep mapping of old *E. coli fadR* mutants accumulated. Molecular dissection of *E. coli* K113 strain, a *fadR* mutant that can grow on decanoic acid (C10) as sole carbon sources unexpectedly revealed a single point mutation of T178G in *fadR* locus (W60G in FadR_k113_). We also observed that a single genetically-recessive mutation of W60G in FadR regulatory protein can lead to loss of its DNA-binding activity, and thereby impair all the regulatory roles in fatty acid metabolisms. Structural analyses of FadR protein indicated that the hydrophobic interaction amongst the three amino acids (W60, F74 and W75) is critical for its DNA-binding ability by maintaining the configuration of its neighboring two β-sheets. Further site-directed mutagenesis analyses demonstrated that the FadR mutants (F74G and/or W75G) do not exhibit the detected DNA-binding activity, validating above structural reasoning.

**Electronic supplementary material:**

The online version of this article (doi:10.1007/s13238-014-0107-3) contains supplementary material, which is available to authorized users.

## Introduction

FadR, a member of the GntR super-family of transcription factors, is recognized as a global regulator (DiRusso et al., 1992, Henry & Cronan, 1992, Clark & Cronan, 2005). Not only does it exerts extensive effects on fatty acid metabolism (Clark, 1981, Nunn et al., 1983, Hughes et al., 1988), but also regulates acetate metabolism (*iclR*) (Gui et al., 1996, Maloy & Nunn, 1981) and stress response (*uspA*) (Farewell et al., 1998). The dual functions of FadR regulatory protein in lipid metabolism can be described as follows: 1) repression of expression of genes essential for fatty acid degradation system, such as *fadBA* (DiRusso et al., 1993, Iram & Cronan, 2006), *fadH* (He et al., 1997, Feng & Cronan, 2010), *fadM* (Feng & Cronan, 2009b), etc.; 2) activation of unsaturated fatty acid biosynthesis pathway via directly up-regulating the transcription of both *fabA* (Nunn et al., 1983, Henry & Cronan, 1992, Campbell & Cronan, 2001) and *fabB* (Campbell & Cronan, 2001, Feng & Cronan, 2009a). The physiological ligands of FadR regulator are long chain fatty acyl-CoA thioesters, which can neutralize and release FadR protein from its binding operators (Henry & Cronan, 1992, Cronan, 1997). Thereby FadR protein can function as a sensor of the availability of long chain fatty acid in bacterial growth environment (Cronan & Subrahmanyam, 1998, DiRusso et al., 1998, Campbell & Cronan, 2001). The phenotype of *E. coli* that wild-type strains cannot grow on decanoic acid (C10) as sole carbon source (only the *fadR* mutant can) is due to the inability of short chain fatty acyl-CoA to turn off FadR-mediated repression of *fad* regulon (Iram & Cronan, 2006).

It is well known that the fatty acid-responsive FadR regulatory protein is exclusively present in γ-proteobacteria (Iram & Cronan, 2005). The paradigm member of FadR regulator is the *E. coli fadR* protein product, a 239-residue polypeptide with solution structure of dimer (van Aalten et al., 2001, Xu et al., 2001, Feng & Cronan, 2010), and also belongs to the helix-turn-helix type transcription factor, comprising N-terminal DNA-binding domain and C-terminal ligand-binding domain (van Aalten et al., 2000, van Aalten et al., 2001, Xu et al., 2001). The accumulated crystal structures of FadR complexed with DNA target/acyl-CoA or alone have established structural basis for FadR-mediated transcription regulation of fatty acid metabolism (van Aalten et al., 2000, van Aalten et al., 2001, Xu et al., 2001). In addition to the crucial residues for ligand binding revealed by biochemical analyses (DiRusso et al., 1998), several amino acids essential for DNA binding have also been implied from FadR structural dissection (Xu et al., 2001). Additionally, the functional diversity has also been suggested by systematically biochemical documentation of a set of bacterial FadR homologues (Iram & Cronan, 2005).

Although the FadR picture seems relatively complete right now, the residues other than direct DNA-binding sites remain unclear that contributes significantly to the binding activity of FadR to target promoters. Systematic analyses of *E. coli fadR* mutants collected in our laboratory in recent years allowed us to unexpectedly discover from K113 strain that a single point mutation (W60G) can impairs dramatically regulatory roles of FadR in lipid metabolism. Structure-guided mutagenesis analyses of FadR protein further suggested that the hydrophobic interaction amongst the three amino acids (W60, F74 and W75) might be essential for its DNA-binding ability by maintaining the configuration of its neighboring two β-sheets. Two more *fadR* super-repressor mutants were also discussed. The missing findings we reported in this paper might add new inputs into the picture of FadR-mediated regulation of bacterial lipid metabolism.

## Results And Discussion

### Phenotypic and genetic dissection of the *fadR* mutant strain K113

**Figure 1:**
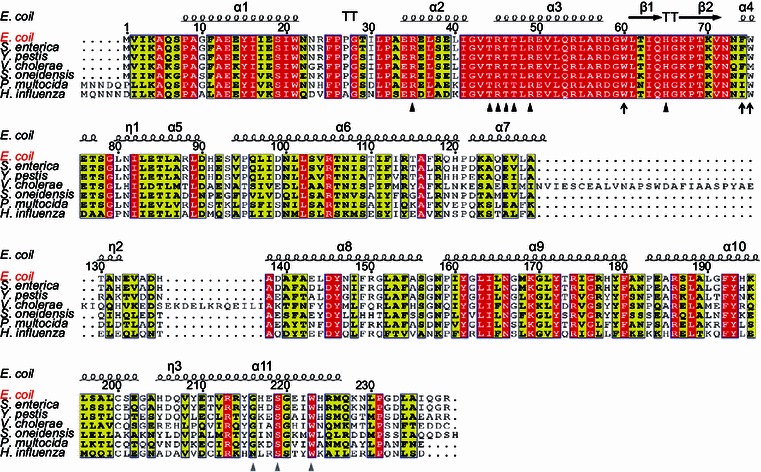
**Sequence alignments of*E. coli*FadR regulatory protein with other homologues from six different species of γ-proteobacteria**. The multiple alignments of amino acid sequences were conducted using ClustalW2 (http://www.ebi.ac.uk/Tools/clustalw2/index.html), and the resultant output was processed by program ESPript 2.2 (http://espript.ibcp.fr/ESPript/cgi-bin/ESPript.cgi), generating the final BLAST photography (Feng & Cronan, 2011b). Identical residues are in white letters with red background, similar residues are in black letters in yellow background, and the varied residues are in grey letters. As we earlier described (Feng et al., 2008), the protein secondary structure was shown in cartoon (on top), in terms of the structural architecture of *E. coli* FadR protein (PDB:1E2X) (van Aalten et al., 2000). α: alpha-helix; β: beta-sheet; T: Turn; η: coil. The DNA binding sites are indicated with black triangles (R35, T44, R45, T46, T47, R49 and 65H) (Xu et al., 2001), the ligand binding sites are shown with grey triangles (216G, 219S and 223W) (van Aalten et al., 2001), and the newly-proposed amino acids essential for DNA binding activity of FadR protein are highlighted with dark arrows (W60, F74 and W75). The FadR sequences are separately sampled from *E. coli* K12 (Accession no.: CAA30881), *S. enterica* (*Salmonella enterica*) (Accession no.: ACF63827), and *Y. pestis* (*Yersinia pestis*) (Accession no.: ZP_06207826), *V. cholerae* (*Vibrio cholerae*) (Accession no.: AAO37924), *S. oneidensis* (*Shewanella oneidensis*) (Accession no.: NP_718457), *P. multocida* (*Pasterurella multocida*) (Accession no.: AAK02132), and *H. influenza* (*Haemophilus influenza*) (Accession no.: AAC22085)

FadR is a well-studied transcription factor that plays multiple roles in regulation of bacterial lipid metabolism. This type of regulator is consisted of a highly-conservative DNA-binding domain and a divergent acyl-CoA ligand-binding motif (Fig. 1). Multiple sequence alignments of a set of FadR homologues revealed its pretty conservative architecture with an exception of *V. cholerae* FadR homologue in that it seemed likely to obtain an extra-inserting sequence of 40 residues (Fig. 1). Earlier biochemical analyses revealed three amino acids at C-terminus of FadR (G216, S219 and 223W) are critical for its binding activity to acyl-CoA ligand (DiRusso et al., 1998), whereas the reported crystal structures visualized six N-terminal residues (R35, 44T, 45R, 46T, 47T, 49R and 65H) in direct contact with its cognate DNA (van Aalten et al., 2000, Xu et al., 2001).

**Figure 2:**
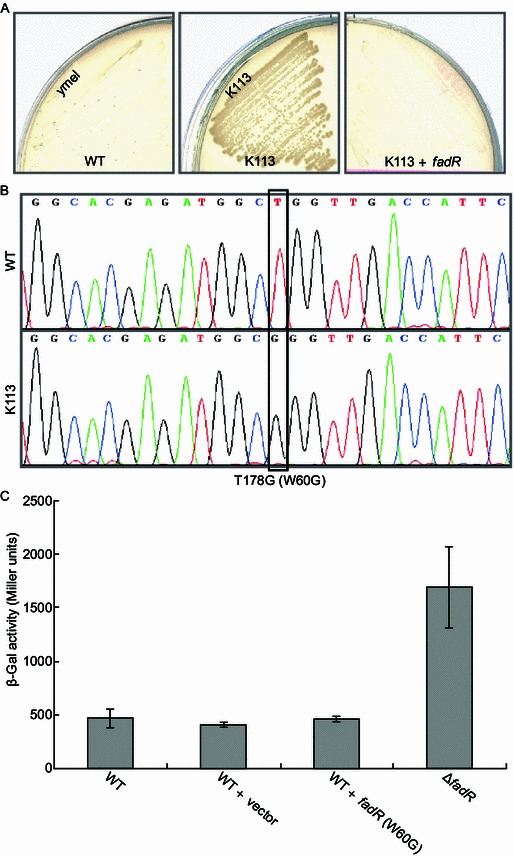
**Determination of a single recessive mutation (W60G) present in*fadR*mutant strain K113**. (A) Growth phenotype of *E. coli fadR* mutant strain K113 on minimal media with C10 as sole carbon source in comparison with those of the wild-type strain and the *fadR* complemented strain. The three strains used here included WT (ymel strain) (Feng & Cronan, 2009a), K113 (*fadR* mutant) (Feng & Cronan, 2009a, Clark et al., 1983) and the complemented strain FYJ7 (K113, *zcf-117*::Tn*10*, *fadR*^+^, Tet^R^) (Feng & Cronan, 2009a). (B) A single mutation (T178G) present in the K113 *fadR* revealed by direct DNA sequencing. The mutation of T178G at DNA level denotes the mutation of at protein level. (C) The mutation of W60G in FadR is genetically recessive. β-Gal activities were recorded in three independent experiments. All the strains assayed here contain a chromosomal *fadBA-lacZ* transcriptional fusion (Table 1). They are SI203 (WT), SI207 (Δ*fadR*), FYJ225 (WT + vector) and FYJ226 (WT + *fadR* (W60G)), respectively. Vector: pBAD24

We had occasion to sequence the original *fadR* mutation that of strain K113 of Overath and coworkers, originally called *dec-16* and now called *fadR16*. We found that the *fadR16* lesion results in substitution of glycine for tryptophan 60, a residue that plays no direct role in DNA binding. We showed that *fadR16* (*fadR*__K113_) was recessive to the wild-type allele. As we knew that the wild-type *E. coli* strains can grow on media with long-chain fatty acids as sole carbon source, but not on short-chain fatty acids like decanoic acid (C10) (Fig. 2A). In contrast, the *fadR* mutants usually exhibited a phenotype with capability of growing on C10 media. Like most of the other *fadR* mutants, the K113 strain can grow on C10 plates (Fig. 2A), and this *fadR* phenotype can be restored to that of the parental strain ymel upon functional complementation of a copy of wild-type *fadR* gene (Fig. 2A). Comparative analyses of direct DNA sequencing results dissected firstly that this *fadR* phenotype is only attributed to the presence of a point mutation of T178G (W60G) in K113 strain (Fig. 2B).

Monitoring β-gal activity of a transcriptional *fadBA-lacZ* fusion revealed that removal of *fadR* led to >3-fold increment of expression level, whereas the introduction of a plasmid pBAD-borne mutant *fadR* (*fadR*_K113_) into the wild-type strain could not result in any significant change of *fadBA* transcription (Fig. 2C). It was anticipated that FadR_K113_ with mutation of W60G cannot compete with the wild-type version of FadR in the context of physiological interplay between FadR and *fadBA* promoter, indicating that the W60G mutation is genetically-recessive.

### Functional impairment of FadR regulatory roles by a genetically recessive single mutation of W60G

**Figure 3:**
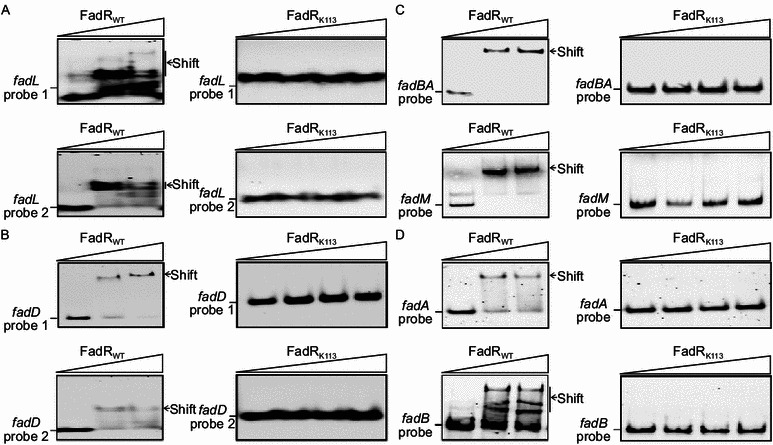
**Mutant FadR protein (FadR**_**K113**_**) losses its DNA binding activity*in vitro***. The two FadR-binding sites from both *fadL* (A) or *fadD* (B) can bind to the wild-type FadR protein (FadR_WT_), whereas not the mutant version of FadR, FadR_k113_ (W60G). (C) The wild-type FadR protein (FadR_WT_) can bind to promoter regions covering the FadR-binding sites of *fadBA* and *fadM*, whereas FadR_k113_ does not. (D) FadR_k113_ fails to bind to promoter regions of *fabA* and *fabB*, two UFA biosynthetic genes whereas FadR_WT_ does. All the EMSA experiments were carried out using 7% native PAGE, and a representative result is shown here. In gel shift assays, FadR_WT_ is added as follows: 0, 0.5 and 1 pmol. Similarly, FadR_K113_ is supplemented in 0, 0.5, 1 and 2 pmol, respectively. All the DIG-labeled probes are added to 0.1 pmol

To fully address the effect of the genetically-recessive single mutation of W60G on regulatory function of FadR protein, we employed the *in vitro* and *in vivo* approaches including EMSA and transcriptional fusion. The DNA binding activity of the mutant FadR protein (FadR_K113_) was examined *in vitro* using gel shift assays. Eight sets of DIG-labeled DNA probes were prepared. Among them, two probes were designed for either *fadL* or *fadD* in that they have two FadR-binding palindromes (*fadL* probe1 plus *fadL* probe 2 (Fig. 3A) and *fadD* probe1 plus *fadD* probe 2 (Fig. 3B)) (Feng & Cronan, 2012). As we expected, the wild-type FadR protein (FadR_WT_) exhibited obvious binding to *fadL* (*fadD*) promoter (Fig. 3A and 3B), however the FadR_K113_ protein was not visualized to interact with them at the same protein level used in our trials (no more than 10 pmol). The promoters of *fadBA* (Feng & Cronan, 2012) and *fadM* (Feng & Cronan, 2009b), two members of β-oxidation system, also cannot be bound by FadR_K113_ protein (Fig. 3C). The similar scenario was also observed with *fabA* and *fabB* (Feng & Cronan, 2011a), two important genes required for UFA biosynthetic pathway (Fig. 3D).

**Figure 4:**
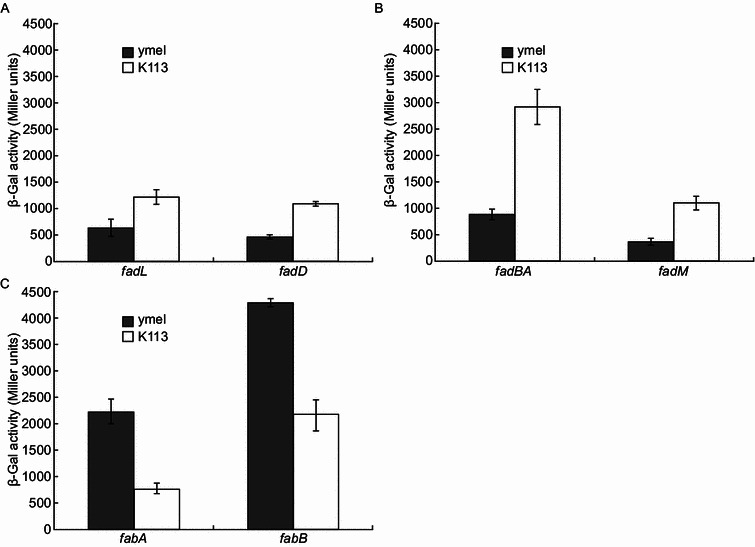
**Regulatory dysfunction of fatty acid metabolism in K113*fadR*strain**. (A) Assays for transcriptional activities *fadL* and *fadD*, two genes of fatty acid transport system in the *fadR* strain K113. (B) Comparative analyses of expression levels of *fadBA,* a major member of beta-oxidation system and *fadM,* an auxiliary player of *fad* system in the *fadR* strain K113 relative to those of the wild-type ymel. (C) Effects on transcriptional levels of *fabA* and *fabB*, two key genes required for UFA synthesis due to *fadR* mutation in K113 strain. *E. coli* strains were grown in RB liquid media. β-Gal activities from three independent experiments are expressed in average ± standard deviations. In panel A, the strains used were FYJ185 (ymel, *fadL-lacZ* fusion), FYJ186 (K113, *fadL-lacZ* fusion), FYJ183 (ymel, *fadD-lacZ* fusion) and FYJ184 (K113, *fadD-lacZ* fusion). In panel B, the four strains included FYJ34 (ymel, *fadBA-lacZ* fusion), FYJ38 (K113, *fadBA-lacZ* fusion), FYJ35 (ymel, *fadM-lacZ* fusion) and FYJ39 (K113, *fadM-lacZ* fusion). In panel C, the strains tested referred to FYJ36 (ymel, *fabA-lacZ* fusion), FYJ 40 (K113, *fabA-lacZ* fusion), FYJ37 (ymel, *fabB-lacZ* fusion), FYJ41 (K113, *fabB-lacZ* fusion), respectively

To further prove the dysfunction of FadR_K113_*in vivo*, we evaluated altered expression profile of a series of FadR-regulated genes in K113 strain vs. ymel strain (parental strain). As a result, we noted that 1) about 2-fold improved expression of both *fadL* and *fadD*, two genes encoding LCFA transport system in K113 strain over ymel strain (Fig. 4A), 2) 3.5-fold increment of *fadBA* expression and 2.5-fold elevated transcription of *fadM* in K113 strain relative to ymel strain (Fig. 4B), and 3) 2 to 2.5-fold decreased expression of *fabA* and *fabB* (Fig. 4C). The combined evidence demonstrated that functional impairment of *fadR*_K113_ regulatory roles in lipid metabolism is due to a genetically-recessive single mutation of W60G in FadR protein.

### A tripartite hydrophobic interaction required for DNA binding activity by FadR

**Figure 5:**
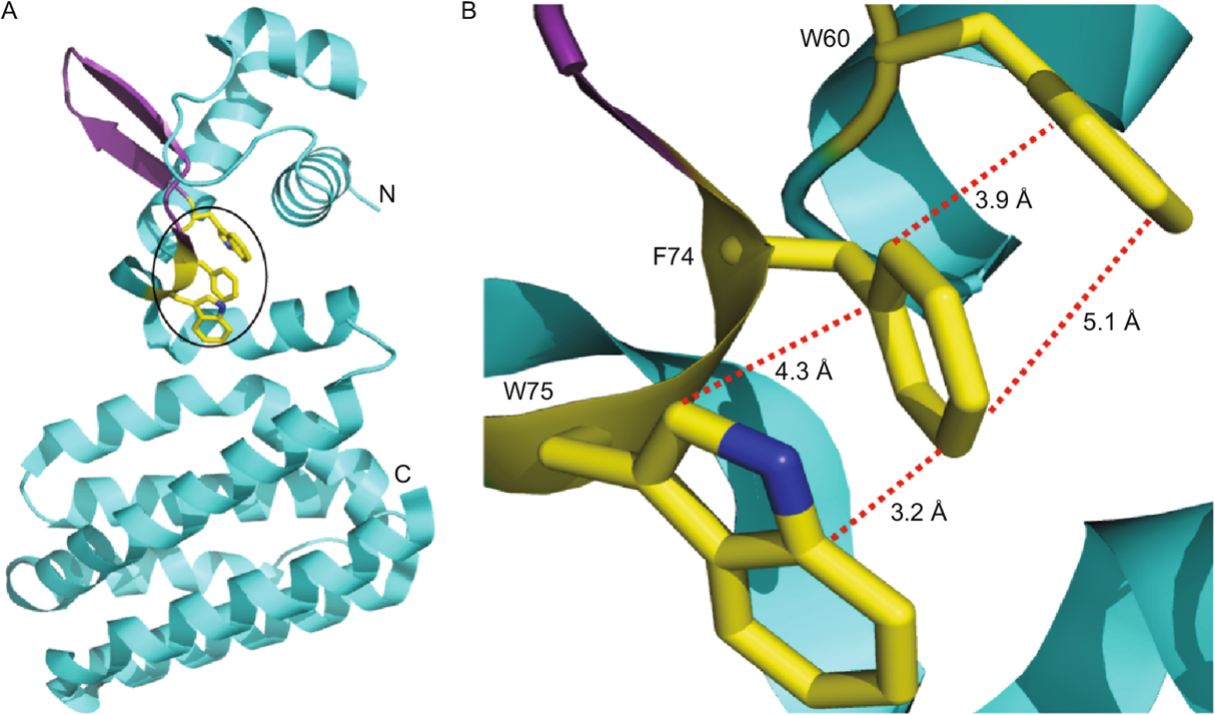
**Structural analyses of FadR protein suggest that a hydrophobic interaction amongst W60, F74 and W75 might be important for its DNA binding activity**. (A) Ribbon structure of *E. coli* FadR in monomer. N: N-terminus; C: C-terminus. α-Helix is blue, β-sheet is purple, and the three amino acids (W60, F74 and W75) that were proposed in this study is highlighted with a circle. (B) The enlarged view of the three critical residues (W60, F74 and W75). The hydrophobic bonds are expressed with dotted red lines, and the instance between two relevant atoms is labeled (angstrom). The photography was generated by Pmol software using the crystal structure’s PDB file of FadR protein (PDB: 1E2X)

Earlier structural investigations illustrated the overall architecture of *E. coli* FadR complex and its target DNA (Fig. S1) (Xu et al., 2001). The integrated evidence from structural modeling combined with genetic/biochemical data showed that the residue W60 is not in direct contact with DNA (Fig. 5A), but rather forms an aromatic cluster with two residues phenylalanine 74 and tryptophan 75 (F74 and W75). The measured distances amongst the three aromatic side chains varied from 3.2 Å to 5.1 Å (Fig. 5B) well positioned for hydrophobic bonding interactions in that it fits generally the criteria for defining a hydrophobic bond (interaction). We ambitiously hypothesize that the above hydrophobic bonds are necessary to maintain the flexibility of the two neighboring β-sheets, which in turn stabilizes configuration of its DNA binding domain (Fig. 5A). If so, mutation of any one of the three residues might well result in the loss of DNA binding activity.

**Figure 6:**
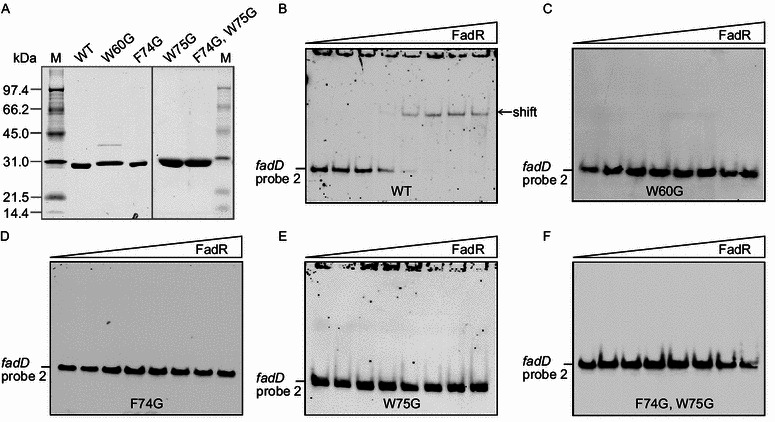
***In vitro*preparation and functional analyses of four mutant FadR proteins plus its wild-type version**. (A) Expression and purification of four mutant FadR proteins and its wild-type. M: protein standard marker (Biorad). EMSA-based assays of binding activities of four mutant FadR proteins, FadR_W60G_ (C), FadR_F74G_ (D), FadR_W75G_ (E) and FadR_F74G, W75G_ (F) in comparison with that of the wild-type version (B). The gel shift tests were all conducted using 7.5% native PAGE, and a representative result is shown here. In these assays, FadR protein is added as follows: 0, 0.05, 0.1, 0.5, 1, 2, 5 and 10 pmol. The DIG-labeled *fadD* probe 2 is added to 0.2 pmol

To test this possibility, we prepared three more mutant FadR proteins (F74G, W75G and F74G plus W75G) using an approach of site-directed mutagenesis. In addition to the two versions of FadR protein (FadR_WT_ and FadR_K113_ (W60G)), we also purified the other three types of mutant FadR proteins to homogeneity (Fig. 6A). Somewhat different from that of the recombinant FadR_WT_ in *E. coli* expression system (>70% solubility), the solubility of all the other four mutant proteins is estimated to vary from 5% to 15% (not shown). The fact that all of the mutants were appreciably less soluble than the wild-type protein is consistent with exposure of hydrophobic groups. For convenient comparison, the DIG-labeled *fadD* probe 2 with relatively high affinity to FadR protein was used in gel shift assays. As we mentioned before, FadR_WT_ protein can bind to *fadD* probe 2 in a dose-dependent manner (Fig. 6B), whereas no significant binding of the mutant protein FadR_K113_ (W60G) was found (Fig. 6C). Similar to what observed with FadR_K113_ mutant protein, other two mutant FadR proteins with single mutation of either F74G (Fig. 6D) or W75G (Fig. 6E) did not exhibit DNA binding activity, as well as the FadR mutant with the double point-mutations of F74G plus W75G (Fig. 6F). Given the above structure-guided site-directed mutational analyses, we concluded that the aromatic cluster plays a key, but indirect, role in DNA binding. The fact that all three residues are aromatic and our prior finding that naturally occurring FadR proteins having isoleucine in place of F74 bind DNA poorly suggested that π-interactions may play a role in cluster function (Xu et al., 2001). However, we found that the F74I version of *E. coli* FadR had full DNA binding activity which ruled out π-interactions. Hence, it seemed likely that the cluster stabilizes the configuration of its DNA binding domain (Fig. 5).

## Conclusions

Although the K113 *fadR* strain has been known for years, we are first to describe systemically regulatory dysfunction of lipid metabolism. Somewhat it is unexpected to note that the molecular determinant is a genetically-recessive point-mutation of W60G in *fadR* locus (Figs. 2 and 5). Relative to the known residues with direct contacting DNA operates, this hydrophobic amino acid of tryptophan at the position 60 of FadR regulatory protein represent a new functional residue in that it is without close contact to its cognate DNA, but required for capability of DNA binding.

In addition, we addressed two more *fadR* super-repressor mutant strains (TH182 and TH183, featuring that the repression of *fad* regulon expression cannot be relieved by oleate). In general consistency with the report by Hughes et al. (Hughes et al., 1988), we also found the molecular determinants of two *fadR* super-repressor strains TH182 and TH183, each of which is a single point mutation localized in C-terminal ligand binding domain (G638A, R213H in TH182 and G647A, G216E in TH183) (not shown). Ambitiously, we firstly proposed a model in which the hydrophobic interaction of W60 with other two aromatic amino acids (F74 and W75) is critical for the maintenance of configuration of the DNA binding domain of FadR protein at N-terminus (Fig. 5). It seemed likely that our findings might supplement three more new functional residues of FadR regulator.

Together, we believed that the deep dissection of the *E. coli* FadR regulatory protein provided a new glimpse of FadR-DNA interplay/crosstalk in the context of lipid metabolism.

## Materials And Methods

### Bacterial strains and growth conditions

All the strains used here are *E. coli* K-12 derivatives (Table 1). Three types of media used for *E. coli* included LB medium (Luria-Bertani medium containing 10 g of tryptone, 5 g of yeast extract and 10 g of NaCl per liter), Rich broth (RB medium containing 10 g of tryptone, 1 g of yeast extract and 5 g of NaCl per liter) and minimal medium M9 (Iram & Cronan, 2006) supplemented with 0.4% glucose, 0.1% vitamin-free Casamino Acids, 1 mmol/L MgSO_4_ and 0.1 mmol/L CaCl_2_, and 0.001% thiamine were used for bacterial growth and analyses of β-galactosidase activity. If necessary, antibiotics were used as follows (in mg/liter): 100 for sodium ampicillin, 50 for kanamycin sulfate, 20 for chloramphenicol and 15 for tetracycline HCl.

**Table 1:** **Strains and plasmids used in this study**

Bacteria or plasmids	Relevant characteristics	References/sources
*E. coli* strains		
BL21 (Tuner)	An expression host for recombinant plasmids	Lab stock
MC4100	F^−^, *araD*139, Δ(*argF-lac*)169	Feng & Cronan (2009b)
DH5α (λ-*pir*)	Δ*lac* host for pAH125 and its derivatives	Haldimann & Wanner (2001); Feng & Cronan (2009b); Feng & Cronan (2009a)
MC1061	Wild-type of *E. coli* K-12, Δ*lac*	Feng & Cronan (2009b)
MFH8	UB1005, *fadR*::Tn*10*	Henry & Cronan (1992)
ymel	Parental strain of K113	Feng & Cronan (2009a)
K113	ymel, *fadR16*	Clark (1981)
JT160	MC1061, *fabA-lacZ* fusion, Kan^R^	Lab stock, Feng & Cronan (2011a)
JT161	MC1061, *fabB-lacZ* fusion, Kan^R^	Lab stock, Feng & Cronan (2011a)
FYJ17	MC1061, *fadM-lacZ* fusion, Kan^R^	Feng & Cronan (2009b)
FYJ30	JT180, *fadR*::Tn*10*, Kan^R^, Tet^R^	Feng & Cronan (2011a)
FYJ32	JT181, *fadR*::Tn*10*, Kan^R^, Tet^R^	Feng & Cronan (2011a)
FYJ34	ymel, *fadBA-lacZ* fusion, Kan^R^	*P1vir*(SI203)xymel^c^
FYJ35	ymel, *fadM-lacZ* fusion, Kan^R^	*P1vir*(FYJ17)xymel^c^
FYJ36	ymel, *fabA-lacZ* fusion, Kan^R^	*P1vir*(JT160)xymel^c^
FYJ37	ymel, *fabB-lacZ* fusion, Kan^R^	*P1vir*(JT161)xymel^c^
FYJ38	K113, *fadBA-lacZ* fusion, Kan^R^	*P1vir*(SI203)xK113^c^
FYJ39	K113, *fadM-lacZ* fusion, Kan^R^	*P1vir*(FYJ17)xK113^c^
FYJ40	K113, *fabA-lacZ* fusion, Kan^R^	*P1vir*(JT160)xK113^c^
FYJ41	K113, *fabB-lacZ* fusion, Kan^R^	*P1vir*(JT161)xK113^c^
SI203	*fadBA-lacZ* transcription fusion	Iram & Cronan (2005); Feng & Cronan (2010)
SI207	*fadBA-lacZ* transcription fusion, Δ*fadR*::Tn*10*	Iram & Cronan (2005); Feng & Cronan (2010)
FYJ104	FYJ103, *fadL-lacZ* transcription fusion	This work
FYJ158	DH5α (λ-*pir*) carrying pAH-*PfadD*	Feng & Cronan (2011a)
FYJ159	MC4100, *fadD-lacZ* transcription fusion integrated into the chromosomal λ-attB site	This work
FYJ183	ymel, *fadD-lacZ* transcriptional fusion	*P1vir*(FYJ159)×ymel^c^
FYJ184	K113, *fadD-lacZ* transcriptional fusion	*P1vir*(FYJ159)× K113^c^
FYJ185	ymel, *fadL-lacZ* transcriptional fusion	*P1vir*(FYJ104)×ymel^c^
FYJ186	K113, *fadL-lacZ* transcriptional fusion	*P1vir*(FYJ104)× K113^c^
FYJ187	MC4100 carrying pINT-ts	Feng & Cronan (2011a)
FYJ191	BL21(DE3) carrying pET28-*fadR*ec(W75G)	This work
FYJ192	BL21(DE3) carrying pET28-*fadR*_K113	This work
FYJ206	BL21(DE3) carrying pET28-*fadR*ec(F74G)	This work
FYJ207	BL21(DE3) carrying pET28-*fadR*ec(F74G/W75G)	This work
FYJ221	Topo 10 carrying pBAD-*fadR*_K113	This work
FYJ225	SI203 carrying pBAD24	This work
FYJ226	SI203 carrying pBAD-*fadR*_K113	This work
Plasmids		
pET28(a)	Commercial T7-driven expression vector	Novagen
pBAD24	Arabinose-inducible promoter-driven expression vector, Amp^R^	Guzman et al. (1995)
pBAD-*fadR*_K113	pBAD24 carrying K113 *fadR* gene, Kan^R^	This work
pET28-*fadR*ec	pET28 carrying *E. coli fadR*, Kan^R^	Feng & Cronan (2009b); Feng & Cronan (2009a)
pET28-*fadR*_K113	pET28 carrying K113 *fadR* gene, Kan^R^	This work
pET28-*fadR*ec(F74G)	pET28 carrying *E. coli fadR* gene with a single F74G mutation, Kan^R^	This work
pET28-*fadR*ec(W75G)	pET28 carrying *E. coli fadR* gene with a single W75G mutation, Kan^R^	This work
pET28-*fadR*ec(F74G, W75G)	pET28 carrying *E. coli fadR* gene with the double mutations (F74G, W75G), Kan^R^	This work

^a^CGSC denotes Coli Genetic Stock Center, Yale University; ^b^ Selection for tetracycline resistance; ^c^ Selection for kanamycin resistance

### Plasmids and DNA manipulations

The expression vector pET28a (Novagen) was used for protein preparation (Table 2). The K113 *fadR* was amplified using primers *fadR*ec-F2 plus *fadR*ec-R2 that carried *Eco*RI and *Sal*I restriction sites, respectively (Table 2). The PCR product was digested with these enzymes and inserted into vector pBAD24 (Guzman et al., 1995) cut with the same enzymes giving the plasmid pBAD-*fadR*_K113 (Table 1). All plasmids acquired were validated by PCR testing plus direct DNA sequencing.

**Table 2:** Primers used in this study

Primers	Primer sequences (5′-3′)
*lacZ-R*	GACCATGATTACGGATTCACTG
*fadR*_K113-F (*Bam*HI)	CG*GGATCC*ATGGTCATTAAGGCGCAAAGCC
*fadR_*K113-R (*Xho*I)	*CCGCTCGAG*TTATCGCCCCTGAATGGCTAAATC
*fadL*-FadR1-F	GCAACATTCC**AGCTGGTCCGACCTATA**CTCTCGCC
*fadL*-FadR1-R	GGCGAGAG**TATAGGTCGGACCAGCT**GGAATGTTGC
*fadL*-FadR2-F	CTCTCGCC**ACTGGTCTGATTTCTAA**GATGTACCTC
*fadL*-FadR2-R	GAGGTACATC**TTAGAAATCAGACCAGT**GGCGAGAG
*fadD*-FadR1-F	GAAACAGC**GGCTGGTCCGCTGTTTC**TGCATTCT
*fadD*-FadR1-R	AGAATGCA**GAAACAGCGGACCAGCC**GCTGTTTC
*fadD*-FadR2-F	GTAATTATCA**AGCTGGTATGATGAGTT**AATATTATG
*fadD*-FadR2-R	CATAATATT**AACTCATCATACCAGCT**TGATAATTAC
*fadBA*-F	ACTTCGACTC**ATCTGGTACGACCAGAT**CACCTTGCGG
*fadBA*-R	CCGCAAGGTG**ATCTGGTCGTACCAGAT**GAGTCGAAGT
*fabA*-F	TTTATTCCG**AACTGATCGGACTTGTT**CAGCGTACACGTGTTAGCTATCCTGCGTGC
*fabA*-R	GCACGCAGGATAGCTAACACGTGTACGCTG**AACAAGTCCGATCAGTT**CGGAATAAA
*fabB*-F	TCTATTAAAT**GGCTGATCGGACTTGTT**CGGCGTACAAGTGTACGCTATTGTGCATTC
*fabB*-R	GAATGCACAATAGCGTACACTTGTACGCCG**AACAAGTCCGATCAGCC**ATTTAATAGA
F74G-F	CGACGAAGGTGAATAATGGCTGGGAAACTTCCGGTT
F74G-R	AACCGGAAGTTTCCCAGCCATTATTCACCTTCGTCG
W75G-F	CGAAGGTGAATAATTTCGGGGAAACTTCCGGTTTAAA
W75G-R	TTTAAACCGGAAGTTTCCCCGAAATTATTCACCTTCG
F74G/W75G-F	CGACGAAGGTGAATAATGGCGGGGAAACTTCCGGTTTAAA
F74G/W75G-R	TTTAAACCGGAAGTTTCCCCGCCATTATTCACCTTCGTCG
pBAD-chk5	CTGTTTCTCCATACCCGTT
pBAD-chk3	GGCTGAAAATCTTCTCT
*fadR*ec-F2 (*Eco*RI)	AACC*GAATTC*ATGGTCATTAAGGCGCAAAGCC
*fadR*ec-R2 (*Sal*I)	CCG*GTCGAC*TTATCGCCCCTGAATGGCTAAATC
T7-F	TAATACGACTCACTATAGGG
T7-R	GCTAGTTATTGCTCAGCGG

The sequences underlined are restriction sites, and the bold letters are putative FadR binding sites

### *P1*_*vir*_ phage transduction

*P1*_*vir*_ transductions were carried out as described by Miller (Miller, 1992) with minor modifications. Strains FYJ34, FYJ35, FYJ36, FYJ37, FYJ183 and FYJ185 (Table 1) were obtained by *P1*_*vir*_ transduction of strain ymel, using lysates grown on strains SI203 (*fadBA-lacZ*), FYJ17 (*fadM-lacZ*), JT160 (*fabA-lacZ*), JT161 (*fabB-lacZ*), FYJ159 (*fadD-lacZ*) or FYJ104 (*fadL-lacZ*) with selection for kanamycin resistance. Strains FYJ38, FYJ39, FYJ40 and FYJ41 were similarly obtained by transduction of strain K113 (Table 1).

### Measurement of β-galactosidase

Mid-log phase cultures in LB, RB or minimal media (with or without supplementation with various carbon sources), were collected by centrifugation, washed twice with Z Buffer (Miller 1972) and assayed for β-galactosidase activity after lysis with sodium dodecyl sulfate-chloroform (Miller 1972). The data were recorded in triplicate with no less than three independent experiments.

### Site-directed mutagenesis

Site-directed mutagenesis was done as we recently described with little changes (Feng et al., 2014). The PCR reaction system (25 μL) consisted of the following components: 2.5 μL of 10× pfx buffer (Invitrogen), 0.5 μL of 40 mmol/L dNTP mix (10 mmol/L each), 1.0 μL of forward/reverse primers (10 pmol/μL), 1.0 μL of pET28-*fadR*ec as template (5 ng/μL), 0.5 μL of Platinum pfx (2.5 U/μL, Invitrogen), and 17.5 μL of distilled sterilized H_2_O. The reaction was performed using the program consisting of a denaturing cycle at 95°C for 5 min; 20 cycles comprised of 95°C for 50 s, 60°C for 50 s, and 68°C for 6 min and a final step of 8 min at 68°C. To remove the residual template plasmid pET28-*fadR*ec, the gel purified PCR products were digested for 1 h with DpnI (20 U/μL, NEB) at 37°C. Subsequently, they were transformed into chemically-competent cells of DH5α and the inserts of purified plasmids were verified by direct DNA sequencing. The plasmids were transformed into BL21 (Tuner) to produce the FadR mutant proteins.

### Protein expression and purification

The hexahistidine-tagged *E. coli* FadR proteins were produced in *E. coli* BL21 (DE3) carrying the appropriate expression plasmids (e.g., pET28-*fadR*ec, Table 1) by induction of bacterial cultures at an OD_600 nm_ of 0.8–1.0 with 0.3 mmol/L IPTG at 30°C for 3 h (Feng & Cronan, 2011b, Feng & Cronan, 2010). The cells were pelleted, washed twice with ice-cold PBS buffer (101.4 mmol/L Na_2_HPO_4_, 1.8 mmol/L KH_2_PO_4_, 137 mmol/L NaCl, 2.7 mmol/L KCl, 8% glycerol, pH7.4), dissolved in the same buffer and lysed using a French Press. The extracts were centrifuged to remove bacterial debris and the supernatants loaded onto a nickel chelate column (Qiagen). Following washing with ten column volumes of PBS buffer containing 50 mmol/L imidazole, the FadR proteins were eluted with 150 mmol/L imidazole. Appropriate eluted protein fractions were pooled and dialyzed against PBS buffer, then concentrated by ultrafiltration (30 kDa cut-off, Amicon Ultra) (Feng & Cronan, 2010). The protein purity was judged by 12% SDS-PAGE, followed by staining with Coomassie brilliant blue R250 (Sigma, St. Louis, MO).

### Electrophoretic mobility shift assays

Electrophoretic mobility shift assays were performed to address interaction between FadR and series of taget DNA probes (e.g., *fadD* and *fadL* (Feng & Cronan, 2012)) essentially as previously reported (Feng & Cronan, 2011b, Feng & Cronan, 2011a, Feng & Cronan, 2009b). With an exception of *fadM* probe (PCR products) (Feng & Cronan, 2009b), all of the FadR-recognizable probes were prepared by annealing two complementary primers (Table 2) by incubation in TEN buffer (10 mmol/L Tris-HCl, 1 mmol/L EDTA, 100 mmol/L NaCl, pH 8.0) at 95°C for 5 min followed by slow cooling to 25°C and then digoxigenin (DIG) labeling by terminal transferase with DIG-ddUTP (Roche). The DIG-labeled DNA probes (either 0.1 pmol or 0.2 pmol) were incubated with either DNA binding protein in binding buffer (Roche) for 15 min at room temperature and then analyzed by native PAGE (7–7.5% PAGE for all probes).

### Bioinformatic analyses

The known FadR sequences were all from the NCBI database and the multiple alignments were done using ClustalW2 (http://www.ebi.ac.uk/Tools/clustalw2/index.html), and the resultant output was processed by program ESPript 2.2 (http://espript.ibcp.fr/ESPript/cgi-bin/ESPript.cgi), generating the final BLAST version. The FadR structure was visualized using PyMol software.

## Electronic supplementary material

Below is the link to the electronic supplementary material.

Supplementary material 1 (PDF 171 kb)
