# Supplementary material for: A new glimpse of FadR-DNA crosstalk revealed by deep dissection of the E. coli FadR regulatory protein
Source: Protein Cell. 2014 Oct 15;5(12):928–39. doi: 10.1007/s13238-014-0107-3 (PMC4259882; doi:10.1007/s13238-014-0107-3)
Supplement: Supplementary file 1 — Supplementary material 1 (PDF 171 kb) [file 13238_2014_107_MOESM1_ESM.pdf]

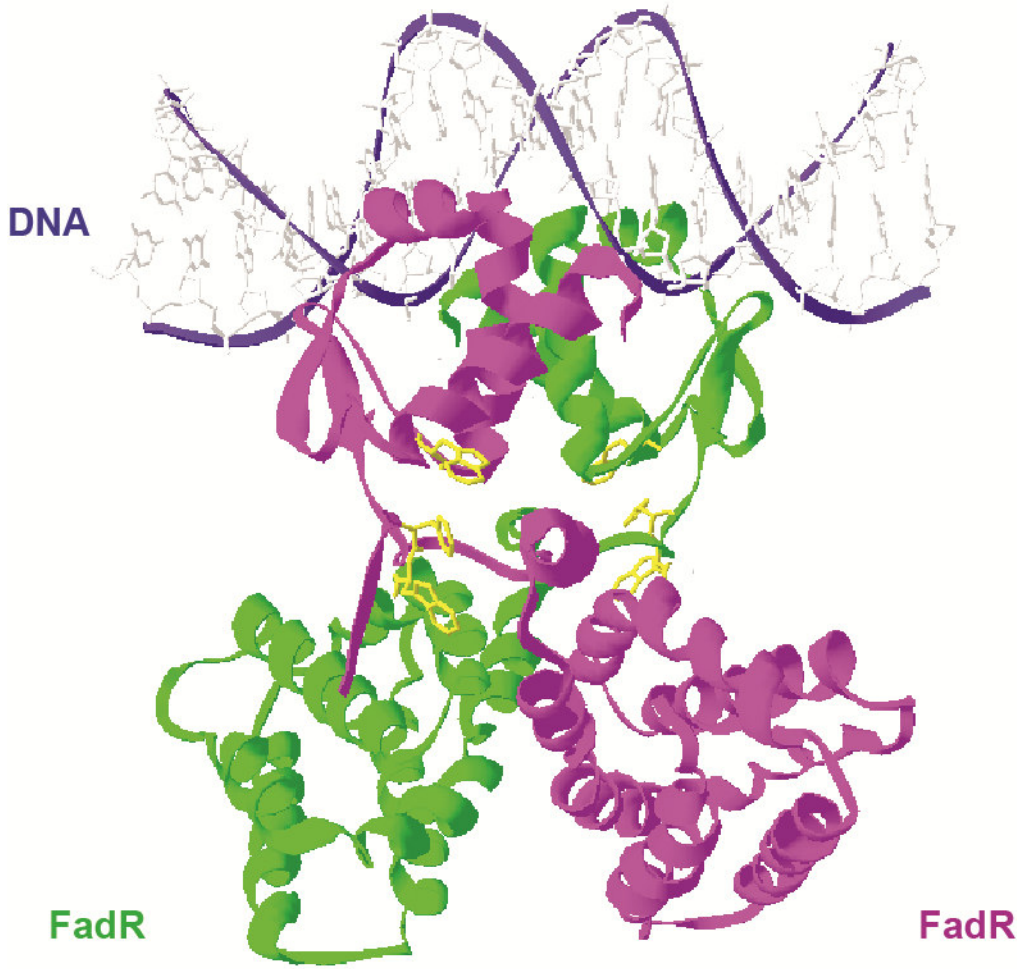

Fig.S1 A snapshot of the *E. coli* FadR protein complexed with its target DNA

DNA helix is denoted in blue.The FadR protein acts in dimer, one of which is in green, and the other is in purple. The putative three residues (W60, F74 and W75) with indirect functions in DNA binding are indicated in yellow. It was generated by Pmol software using the crystal structure of *E. coli* FadR protein (PDB:1H9T).
